# Bone: the final frontier for *Staphylococcus aureus* penetration in chronic rhinosinusitis

**DOI:** 10.1186/1916-0216-42-45

**Published:** 2013-07-19

**Authors:** Rogério Pezato, Luciano Bottura, Rodrigo de Paula Santos, Richard Louis Voegels, Andre Luis Lacerda Bachi, Luis Carlos Gregório

**Affiliations:** 1 Department of Otorhinolaryngology, University of São Paulo, São Paulo, Brazil; 2 Military Police Hospital, São Paulo State Military Police, São Paulo, Brazil; 3 Department of Otorhinolaryngology, Federal University of São Paulo - Unifesp, São Paulo, Brazil; 4 Department of Immunology, Federal University of São Paulo - Unifesp, São Paulo, Brazil

**Keywords:** Rhinosinusitis, Bone, Staphylococcus aureus, Airway

## Abstract

The superantigenic properties of *Staphylococcus aureus* have been implicated in increasing the inflammatory process in airway diseases. Local formation of IgE antibodies against staphylococcal enterotoxins by secondary lymphoid tissue in nasal polyps has been demonstrated. *Staphylococcus aureus* is known to colonize the nasal mucosa, and has been found invading the nasal submucosa and intracellularly.

**Objective:**

To evaluate the limits of *Staphylococcus aureus* invasion in the upper airway.

**Material and methods:**

Inferior turbinate samples from 3 patients without sinus disease, 6 ethmoid samples from patients with chronic rhinosinusitis with nasal polyposis, and 6 ethmoid samples from patients with chronic rhinosinusitis without nasal polyposis were studied. A fluorescein-labeled PNA probe against *Staphylococcus aureus* was used to test for the presence of the bacterium in bone (after decalcification) and mucosa.

**Results:**

We found *Staphylococcus aureus* invading the nasal submucosa in patients with nasal polyposis, but no cases of *Staphylococcus aureus* positivity in bone. In conclusion, we cannot support the hypothesis of nasal bone as a reservoir for *Staphylococcus aureus*, releasing massive amounts of staphylococcal enterotoxins and eliciting an inflammatory reaction, as occurs with the nasal mucosa.

## Introduction

The superantigenic properties of *Staphylococcus aureus* (SA) have been implicated in increasing the inflammatory process and modifying immune cell behavior in airway disease. Specifically, studies of patients with chronic rhinosinusitis with nasal polyposis (CRSwNP) studies support the role of *Staphylococcus aureus* enterotoxins (SE) in shifting the Th balance (Th1/Th2) to a Th2 inflammatory response [1,2].

Furthermore, there is clear evidence that IgE and specific IgE antibodies against SE (SE-IgE) are found in higher levels in the nasal tissues of patients with CRSwNP as compared with healthy subjects or patients with chronic rhinosinusitis without nasal polyposis (CRSsNP) [3]. SE-IgE expression by secondary lymphoid tissue in nasal polyps has been demonstrated elsewhere [4].

SA has long been described as one of the microorganisms most commonly isolated from the sinuses of chronic rhinosinusitis patients [5]. Interestingly, SA also colonizes the nasal cavity of healthy subjects, and some authors have reported no difference in the SA colonization rate between controls and patients with nasal polyposis [6-8].

The link between SE-IgE and the presence of SA is not entirely clear; indeed, a subset of SE-IgE positive patients with CRSwNP who are not colonized by SA has been described [4].

In an attempt to better understand the linkage between SA and the SE-IgE immune response in patients with CRSwNP, possible reservoirs for SA have been evaluated. Such reservoirs could release massive amounts of SE, eliciting an inflammatory reaction via their superantigenic properties and thus resulting in polyclonal activation of T- and B-lymphocytes, SE-IgE production, and perpetuation of the inflammatory process.

A biofilm can be defined as a group of adherent bacteria irreversibly anchored to a surface and enclosed in a matrix of exopolysaccharides [9]. SA biofilms are commonly found in nasal polyps as a nidus from which SE can be released into the paranasal sinuses, and their presence is associated with eosinophilic inflammation and high levels of IL-5 and ECP [10].

Although increases in polyclonal IgE production and SE-IgE have been demonstrated in CRSwNP, whether SA biofilms are more common in CRSwNP patients as compared with health subjects or CRSsNP patients remains unclear [11]*.*

Recent studies have demonstrated the presence of SA not only in the lining of the nasal epithelium, but also in the nasal submucosa [12], suggesting that SA invasion occurs preferentially in CRSwNP-affected mucosa as compared with healthy mucosa or mucosa affected by CRSsNP [13]. Corroborating these findings, some authors have suggested that SA may be present intracellular [13,14].

Due the importance of the anti-SA immune reaction in the pathogenesis of CRS and its unclear association with actual presence of SA, this study set out to evaluate whether bone could serve as a reservoir for *Staphylococcus aureus*, thus providing a complete picture of staphylococcal invasion of the nasal cavity.

## Material and methods

### Patients

Patients were recruited from the Departments of Otorhinolaryngology of Ghent University Hospital, Ghent, Belgium, and the Federal University of São Paulo, São Paulo, Brazil.

Inferior turbinate samples from adult patients without sinus disease undergoing septoplasty were collected and used as controls (n = 3). Ethmoid samples from adult patients with CRSwNP (n = 6) and without nasal polyposis (CRSsNP) (n = 6) were obtained during functional endoscopic sinus surgery (FESS) procedures. The diagnosis of sinus disease was based on history, clinical examination, nasal endoscopy and computed tomography of the paranasal cavities, according to EPOS guidelines [15]. The study was approved by the Research Ethics Committee of the Federal University of Sao Paulo (protocol no.: 06808412.2.0000.5505), and written informed consent was obtained from each patient before specimen collection.

### Tissue preservation and preparation for staining

Inferior turbinate mucosa and inferior turbinate bone from healthy subjects and ethmoidal mucosa and ethmoid bone from CRSwNP and CRSsNP patients were placed in 10% acetaldehyde immediately after surgical removal and kept for 24 hours at room temperature.

The specimens were then preserved in 70% ethanol at 4°C, embedded in paraffin, and cut into 4-μm-thick sections with a microtome. Sections were then affixed onto Superfrost Plus glass slides (Menzel Glaser, Braunschweig, Germany). Once mounted, the slides were dried at 60°C for a few hours.

### Bone decalcification

A solution of 10% ethylenediamine tetraacetic acid (EDTA) in distilled water, pH 7.4, was used to remove the calcium from all bone specimens before preservation in 70% ethanol. The specimens were kept in this solution under continuous stirring, at 4°C, over several days (mean 10 days), depending on size and degree of mineralization. The chelating solution (EDTA 10%) was changed once a day.

### Hematoxylin and eosin staining

After decalcification, sections were rinsed in distilled water. The nuclei were stained with alum hematoxylin (Lillie–Mayer’s solution) for 5 minutes and rinsed in running tap water. Differentiation was performed with 0.3% acid alcohol and sections were rinsed again in running tap water and, subsequently, in Scott’s tap water substitute (sodium hydrogen carbonate 10 g, magnesium sulphate 100 g, distilled water 5 L).

After rinsing in tap water, the sections were stained with eosin solution (1% eosin Y 400 mL, 1% aq. phloxine 40 mL, 95% alcohol 3100 mL, and glacial acetic acid 16 mL) for 2 minutes, dehydrated, and cleared.

### Immunohistochemical staining

For deparaffinization, slides were washed successively in xylene (3 times for 10 minutes), 100% ethanol (2 times for 5 minutes), 90% ethanol (2 times for 5 minutes), and 70% ethanol (2 times for 5 minutes).

After air-drying, the sections were hybridized at 55°C for 90 minutes in a humidified chamber with 100–500 NM of fluorescein-labeled PNA probe (*S. aureus*/CNS PNA-Fish kit; LOT 03912A-US, AdvanDx, Woburn, MA, USA). After hybridization, the coverslips were removed by submerging each slide in the wash buffer provided with the kit and washed for 30 minutes in a bath at 50°C.

Each slide was mounted on Vectashield (Vector, Burlingame, CA) containing 4,6-diamidino-2-phenylindole dihydrochloride (Roche Molecular Biochemicals, Brussels, Belgium) to counterstain the nuclei.

**Figure 1:**
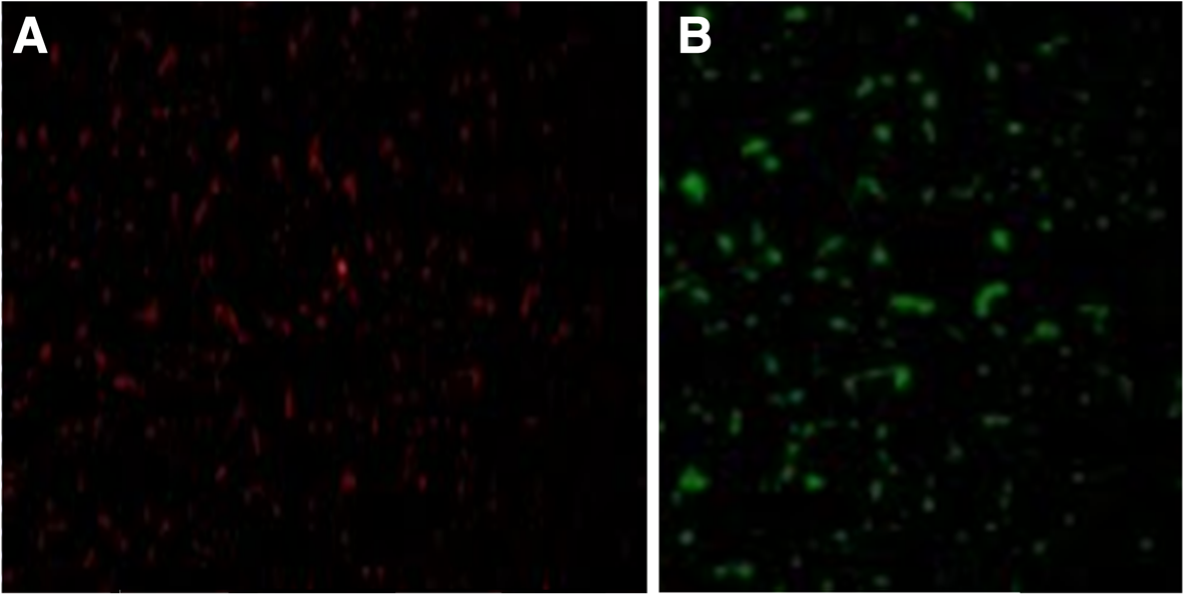
**Positive control. A**, *S. epidermidis* (red); **B**, *S. aureus* (green).

The positive control well contained *Staphylococcus aureus* and *Staphylococcus epidermidis* to confirm the high specificity of the probe to distinguish SA from coagulase-negative staphylococci. SA should test green-positive, whereas *S. epidermidis* should test red-positive (*S. aureus*/CNS Control Slides kit; LOT QST-0099-US, AdvanDx, Woburn, MA, USA, Figure 1). The negative control well contained *S. agalactiae.*

Microscopic examination was conducted with a Zeiss Axioplan epifluorescence microscope (Carl Zeiss, Gottingen, Germany) equipped with a CCD camera (IMACCCD S30; SONY, Germany) using a fluorescein isothiocyanate-specific filter. Images were captured using the Isis imaging and software system (MetaSystems; Sandhausen, Germany).

### Statistics

The data generated in the study were analyzed in SPSS 18 (IBM Corporation, NY, USA). Fisher’s exact test was used to evaluate between-group differences in two categorical variables. *P*-values of less than 0.05 were considered significant.

## Results

### Hematoxylin and Eosin Staining

**Figure 2:**
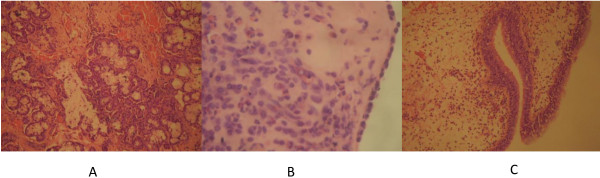
**Nasal mucosa stained with hematoxylin and eosin. A**, demonstrates mucus-secreting glands, hemorrhagic foci and lymphocytic infiltration in the mucosa of the inferior turbinate of a healthy subject (×400 magnification); **B**, lymphoplasmacytic and eosinophil infiltration of the ethmoidal mucosa in a patient with CRSsNP (×400 magnification); **C**, inset, pseudostratified columnar epithelium with edema of the lamina propria and lymphocytic and eosinophil infiltration of a nasal polyp in a patient with CRSwNP (×100 magnification).

**Figure 3:**
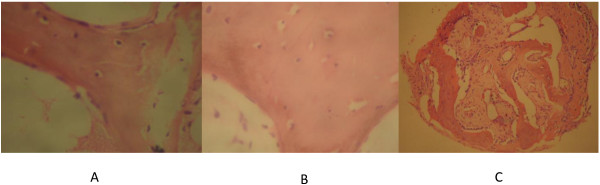
**Bone stained with hematoxylin and eosin. A**, shows preserved osteocytes surrounded by bone matrix in the inferior turbinate of a healthy subject (×400 magnification); **B**, preserved osteocytes preserved surrounded by bone matrix in the ethmoid bone of a patient with CRSsNP (×400 magnification); **C**, preserved osteocytes surrounded by bone matrix in the bone of a patient with CRSwNP (×100 magnification).

Hematoxylin and eosin (H&E) staining was used to assess the presence of cells in mucosa specimens and, especially, in the bone specimens after decalcification. The H&E stain demonstrated epithelium in the nasal mucosa and submucosa of our samples (Figure 2), and bone cells in the decalcified ethmoid bone and inferior turbinate specimens (Figure 3).

### PNA-fish

**Figure 4:**
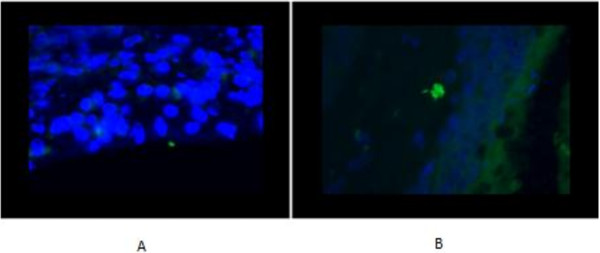
**Positive staining (in green) for presence of *Staphylococcus aureus *in the nasal mucosa. A**) on the epithelium, **B**) intramucosal.

Out of a total of 15 subjects (Table 1), we identified SA in the mucosa of 2 patients with CRSwNP (33%). In one patient, a cluster of SA was visible on the nasal epithelium, and in the other, a cluster of SA was detected in the submucosa (Figure 4). We did not find SA in the mucosa of controls or patients with CRSsNP. The presence of SA in the mucosa of CRSwNP patients was not statistically significant compared to CRSsNP patients and controls.

**Table 1:** **Prevalence of *Staphylococcus aureus *in the bone and nasal mucosa according to diagnosis**

**Patient**	**Diagnosis**	**Presence of SA**	**Presence of**
**identification**		**in the mucosa**	**SA in bone**
01	CRSwNP	Positive	Negative
02	CRSwNP	Positive	Negative
03	CRSwNP	Negative	Negative
04	CRSwNP	Negative	Negative
05	CRSwNP	Negative	Negative
06	CRSwNP	Negative	Negative
07	Control	Negative	Negative
08	Control	Negative	Negative
09	Control	Negative	Negative
10	CRSsNP	Negative	Negative
11	CRSsNP	Negative	Negative
12	CRSsNP	Negative	Negative
13	CRSsNP	Negative	Negative
14	CRSsNP	Negative	Negative
15	CRSsNP	Negative	Negative

**Figure 5:**
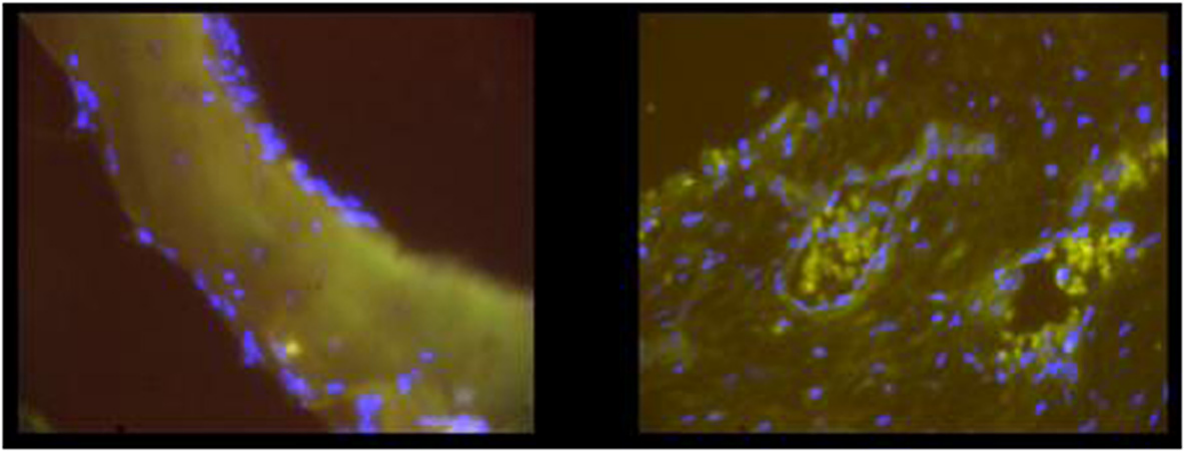
**Absence of staining for *Staphylococcus aureus *in the bone.**

We did not detect SA in any bone specimens (0 out of 15), regardless of diagnosis or group (Figure 5).

## Discussion

The role of *Staphylococcus aureus* acting as a superantigen in CRS has been well demonstrated [1,16]_,_ as has the unquestionable correlation between SE-IgE levels and CRSwNP [3]. Based on these findings, it would be reasonable to presume that the prevalence of SA is higher in the microbiology of patients with CRSwNP as compared with healthy subjects or patients with CRSsNP, but many studies have failed to show any difference in sinus microbiology between CRSwNP and CRSsNP [6-8]. Furthermore, complex forms of colonization, such as SA biofilms in the sinus mucosa, are not clearly associated with CRSwNP [11].

In an attempt to explain the high levels of SE-IgE and the presence of secondary lymphoid tissue found locally in nasal polyp tissue, research has been conducted into possible reservoirs that could act as a continuous source of SE and, consequently, perpetuate a severe inflammatory process.

SA penetration into the nasal mucosa has been confirmed [12,13] and demonstrated to be higher in CRSwNP patients than in controls and patients with CRSsNP. Corroborating these findings, our study found SA only in the mucosa of patients with CRSwNP.

The main novel contribution of this study was the search for SA beyond the sinus mucosa; namely, in bone.

Evaluation of bone tissue is still challenging due to its mineralization. The chosen chelating agent was EDTA, as it is suitable for immunohistochemical staining, because it better preserves bone matrix proteins and genetic material for future analysis [17]. Hematoxylin and eosin staining played an important role in confirming that the decalcification protocol used did not affect the presence of osteocytes.

In view of our findings, we suggest that, unlike nasal mucosa, bone does not play a role as a reservoir for SA. Nevertheless, other studies with larger samples and alternative techniques are required to confirm our results.

## Competing interests

None of the authors report any conflict of interest.

## Authors’ contributions

RP was involved in all stages of the study. LB, ALLB, and RPS were involved in data collection and immunohistochemical staining. RLV and LCG were involved in drafting the manuscript and revising it critically. All authors gave final approval for the publication of this manuscript. All authors read and approved the final manuscript.
